# Bone morphogenetic protein 9 enhances osteogenic and angiogenic responses of human amniotic mesenchymal stem cells cocultured with umbilical vein endothelial cells through the PI3K/AKT/m-TOR signaling pathway

**DOI:** 10.18632/aging.203718

**Published:** 2021-11-27

**Authors:** Ziming Liu, Yuwan Li, Jianye Yang, Jiaxing Huang, Changqi Luo, Jun Zhang, Wenqiang Yan, Yingfang Ao

**Affiliations:** 1 Department of Sports Medicine, Peking University Third Hospital, Institute of Sports Medicine of Peking University, Beijing Key Laboratory of Sports Injuries, Beijing 100191, China; 2 Department of Orthopaedics, The First Affiliated Hospital of Zunyi Medical University, Zunyi 563000, Guizhou, China; 3 Department of Orthopaedics, The First Affiliated Hospital of Chongqing Medical University, Chongqing 400042, China; 4 Department of Orthopaedics, The Second People’s Hospital of Yibin, Yibin 644000, Sichuan, China

**Keywords:** bone morphogenetic protein 9, human amniotic mesenchymal stem cells, umbilical vein endothelial cells, PI3K/AKT/m-TOR signaling pathway

## Abstract

Background: Neovascularization plays an essential part in bone fracture and defect healing, constructing tissue engineered bone that targets bone regeneration. Bone morphogenetic protein 9 (BMP9) is a regular indicator that potentiates osteogenic and angiogenic differentiation of MSCs.

Objectives: To investigate the effects of BMP9 on osteogenesis and angiogenesis of human amniotic mesenchymal stem cells (hAMSCs) cocultured with human umbilical vein endothelial cells (HUVECs) and determine the possible underlying molecular mechanism.

Results: The isolated hAMSCs expressed surface markers of MSCs. hAMSCs cocultured with HUVECs enhance osteogenic differentiation and upregulate the expression of angiogenic factors. BMP9 not only potentiates angiogenic signaling of hAMSCs cocultured with HUVECs also increases ectopic bone formation and subcutaneous vessel invasion. Mechanically, the coupling effect between osteogenesis and angiogenesis induced by BMP9 was activated by the BMP/Smad and PI3K/AKT/m-TOR signaling pathways.

Conclusions: BMP9-enhanced osteoblastic and angiogenic differentiation in cocultivation with hAMSCs and HUVECs *in vitro* and *in vivo* also provide a chance to harness the BMP9-regulated coordinated effect between osteogenic and angiogenic pathways through BMP/Smad and PI3K/AKT/m-TOR signalings.

Materials and Methods: The ALP and Alizarin Red S staining assay to determine the effects of osteoblastic differentiation. RT-qPCR and western blot was measured the expression of angiogenesis-related factors. Ectopic bone formation was established and retrieved bony masses were subjected to histochemical staining. The angiogenesis ability and vessel invasion were subsequently determined by immunofluorescence staining. Molecular mechanisms such as the BMP/Smad and PI3K/AKT/m-TOR signaling pathways were detected by ELISA and western blot analysis.

## INTRODUCTION

Bone formation and rehabilitation are successive processes that in possession of highly coordinated interactions in different type of cells and tissues to form new mineralized tissues, including vascular endothelium and autonomic or sensory nerves; the process is primarily mediated by osteoblasts [1, 2]. Blood vessels are structural templates for bone development that aggregate key substances of bone homeostasis into the osteoblastic micro-environment, including mineral salts, growth factors, and osteoblastic related cells [3]. Mesenchymal stem cells (MSCs) possess multiple differentiation potential, such as osteogenic, chondrogenic, and adipogenic lineages [4, 5]. The human amniotic mesenchymal stem cells (hAMSCs) have become popular since it is a highly vascular membrane, with noninvasive isolation from an unlimited source and lacks ethical controversy. hAMSCs have been tremendously used in bone surgeries, including spine surgeries and in sports medicine [6–8]. Due to its advantageous properties of favorable proliferation, migration, and immunomodulation, hAMSCs are becoming clinically promising [9, 10]. Most if not all osteogenic differentiation is a continuous waterfall that reproduces molecular events that occur during embryonic bone development [11, 12]. Bone morphogenetic proteins (BMPs) exert a vital role in the development of bone formation and rehabilitation, which have shown to enhance proliferation and osteogenic and angiogenic differentiation of stem cells [13, 14]. They be part of the transforming growth factor-β super family, and rodents have been discovered [15, 16].

Upon osteogenic activity analysis, we found that BMP9 is most latent capacity among the 14 types of BMPs in the induction of osteoblastic differentiation of MSCs *in vitro* and *in vivo* [17, 18]. BMP9, as one of the least studied BMPs can regulating endothelial cell function and angiogenesis, inducing choline-like phenotype of embryonic basal precholinergic neurons which possess restraining the production of hepatic glucose [19, 20]. Through transcriptional profiling, we demonstrate that BMP9 regulates a diverse set of downstream target genes in MSCs and cross-talk with other pathways as well. As one of the hot part of BMPs, BMP9-induced osteoblastic differentiation of MSCs still need to be fully illuminated.

Studies have shown that angiogenesis precedes bone formation, and the bone vessel system not only provides the necessary nutrients, growth factors, hormones, cytokines for bone formation, also removes metabolic waste, acts as a bridge between bone and surrounding tissue [21, 22]. Therefore, the "ternary regulation theory" of bone metabolism was put forward which includes angiogenesis, osteogenesis and bone resorption with osteoclasts [23, 24]. Angiogenesis may provide a new target for prevention and treatment of bone mass loss. MSCs could differentiate into other cell types, originating from a mesoblast, in appropriate culture condition. Among these cells, endothelial cells (ECs) can express some cytokines induce osteogenic differentiation when exposed to MSCs [25–28]. Therefore, the application of co-culture of MSCs and endothelial cells to expand the number of MSCs and promote angiogenesis and osteogenesis may provide a promising method for bone tissue engineering and bone regeneration. However, the specific mechanism of cell co-culture has not been fully studied.

Here, the objectives of this study were to: (1) investigate whether hAMSCs cocultured with human umbilical vein endothelial cells (HUVECs) enhance osteogenic differentiation; (2) determine whether BMP9 can enhance osteogenic and angiogenic differentiation of hAMSCs cocultured with HUVECs *in vitro* and *in vivo*; (3) investigate the molecular mechanism underlying BMP9 functions in hAMSCs cocultured with HUVECs.

## RESULTS

### Characterization of hAMSCs and HUVECs

**Figure 1:**
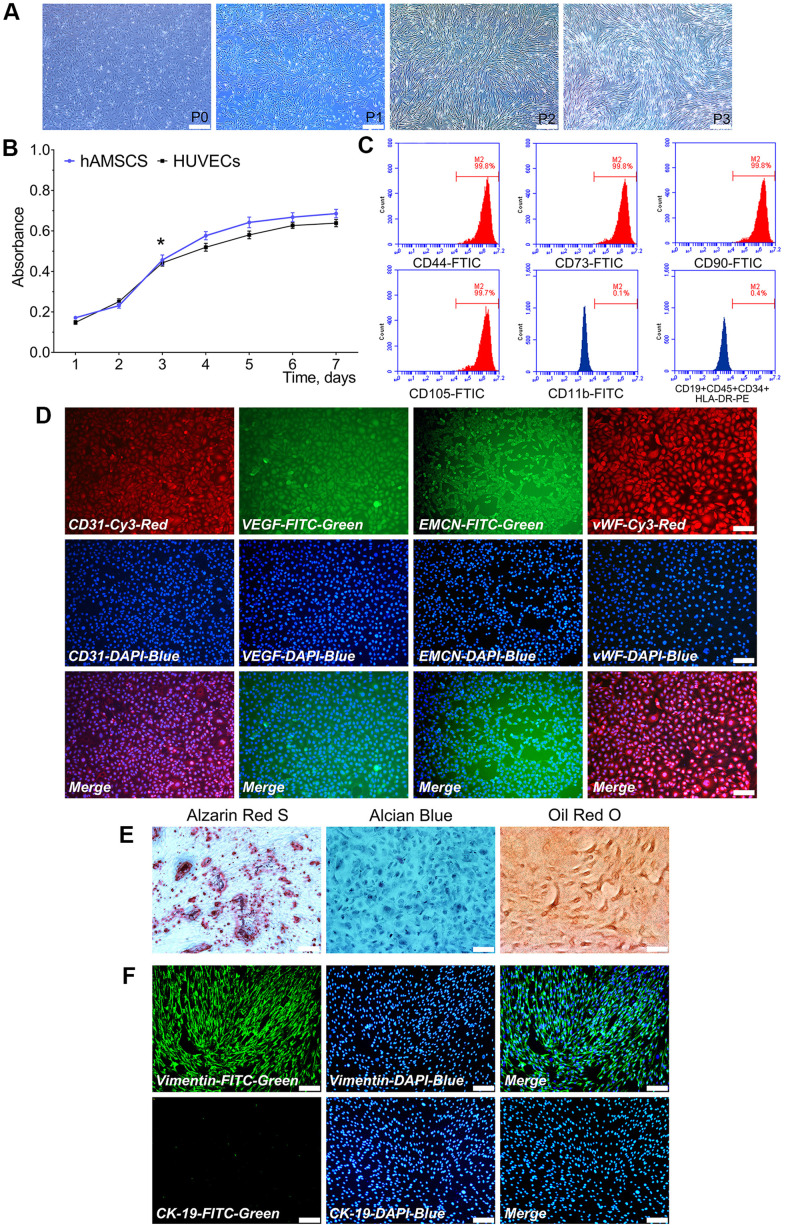
**Characterization of isolated hAMSCs and HUVECs.** hAMSCs at P0 to P3 showing a fibroblast-like morphology and spindle shaped configuration (×40, bar: 200 μm) (**A**); CCK-8 assay showed the doubling time for hAMSCs and HUVECs was 28 h and 33.5 h, respectively (*P < 0.05) (**B**); Flow cytometry results showed phenotypical identities of hAMSCs at P3 (**C**); Phenotypic properties of HUVECs and immunofluorescence staining showed HUVECs at P3 highly expressed the surface markers of endothelial cells (×100, bar: 50 μm) (**D**); Multi-lineage differentiation potential of hAMSCs *in vitro*. Alizarin Red S staining of hAMSCs for 21 d. Alcian Blue staining of hAMSCs for 21 d. Oil Red O staining of hAMSCs for 21 d (×100, bar: 50 μm) (**E**); hAMSCs at P3 scarcely expressed CK-19 and highly expressed vimentin. CK-19 and vimentin were stained green by FTIC, and cell nuclei were stained blue by 4’, 6-diamidino-2-phenylindole (DAPI) (×100, bar: 50μm) (**F**). FITC: Fluorescein isothiocyanate; PE: Phycoerythrin.

Primary cultured hAMSCs adhered to the culture flask within 24 h and its morphology observed after 24 h. hAMSCs at P1, P2, and P3 exhibited a spindle-shaped extrinsic feature. During primary culture, hAMSCs reached 80-90% confluence at 5 d (Figure 1A). Proliferation ability of hAMSCs and HUVECs were determined by the CKK-8 assay. The hAMSCs and HUVECs went through a logarithmic phase for 2-3 d after 1 d of incubation period, where the multiplication rate was recorded. The doubling times of the HUVECs and hAMSCs were 33.5 h and 28 h, respectively (Figure 1B). Flow cytometry (FCM) assay showed that hAMSCs, positively expressed specific surface markers of MSCs including CD73, CD90, CD105, and CD44, while negatively possessed markers CD19, CD45, CD34, and HLA-DR at P3 (Figure 1C). The immunofluorescence staining assay showed that HUVECs greatly expressed angiogenic relative markers CD31, VEGF, Endomucin (EMCN), and vWF (Figure 1D). Moreover, multiple-directional differentiation of hAMSCs showed that isolated cells be able to differentiated into osteoblastic, chondrogenic and adipogenic cells after being cultured in specifically appointed differentiation culture medium at 21 d (Figure 1E). In the isolation procedures of hAMSCs, the human amniotic epithelial cells (hAECs) are interfused with hAMSCs. In hAECs, CK-19 exists as a specific marker and vimentin exists as a specific marker in hAMSCs. The immunofluorescence staining assay revealed that hAMSCs positively expressed vimentin which negatively expressed CK-19 at P3 (Figure 1F).

### BMP9 enhances osteoblastic differentiation of cocultures of hAMSCs and HUVECs

**Figure 2:**
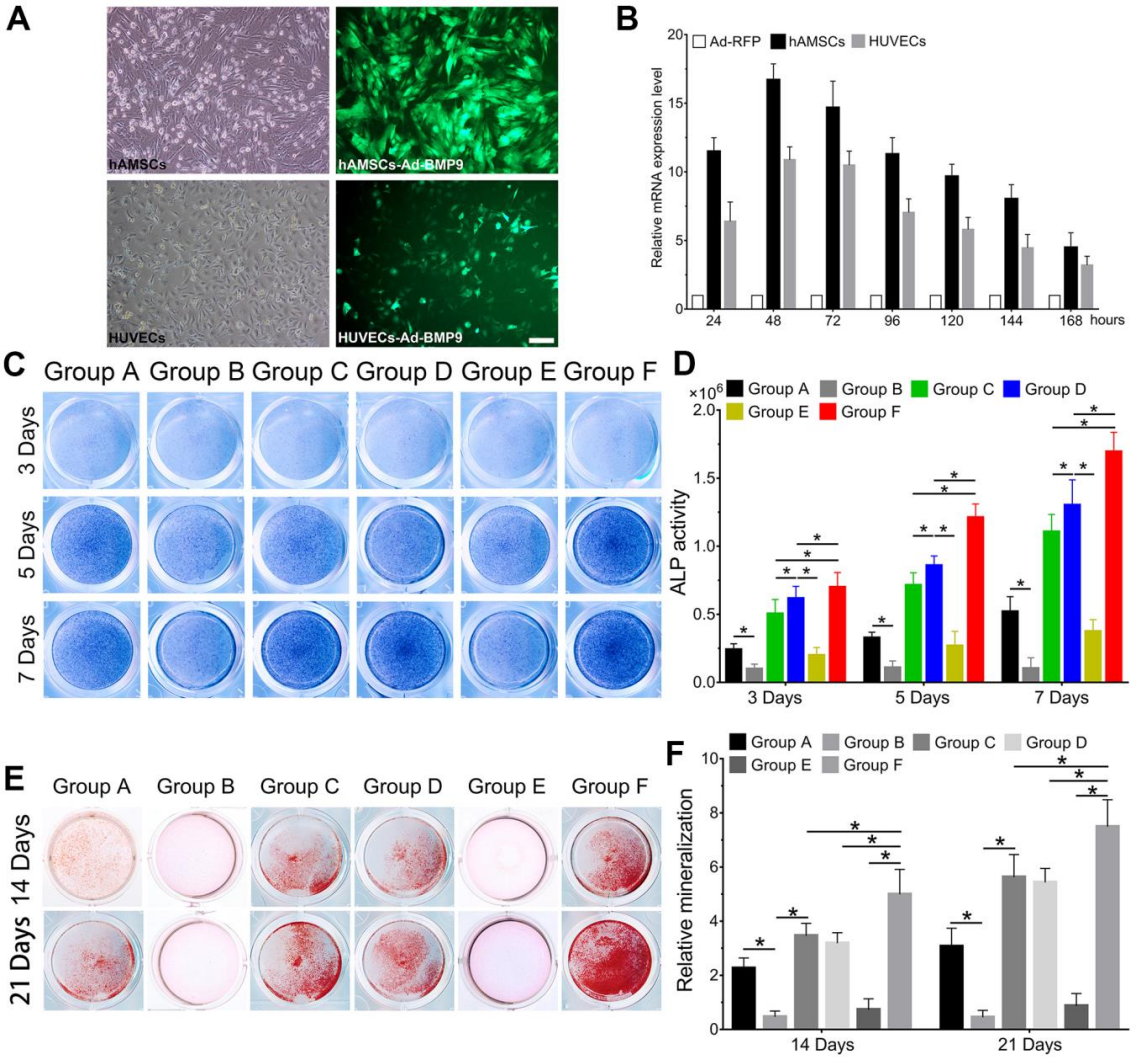
**BMP9 enhances osteoblastic differentiation of hAMSCs cocultured with HUVECs *in vitro*.** The adenovirus Ad-BMP9 (green) was shown to successfully transfect hAMSCs and HUVECs for 24 h (×100, bar: 50μm) (**A**); Ad-BMP9 upregulated the gene expression level of BMP9 over 17 times and 12 times of hAMSCs and HUVECs at 48 h, respectively (**B**); Overexpressed BMP9 enhanced ALP activity in hAMSCs cocultured with HUVECs at 3, 5 and 7 d (**C**); ALP biochemical quantification results showed BMP9 increased the expression of ALP in cocultured hAMSCs and HUVECs at 3, 5 and 7 d, respectively (**D**); The bone mineralization in each group were detected by Alizarin Red S staining assay under the treatment as shown at 14 and 21 d (**E**); The quantification of mineralization in each group were analyzed under the treatment as shown at 14 and 21 d (**F**) (*P < 0.05).

We observed the effect of cocultures of hAMSCs and HUVECs on BMP9-induced osteoblastic differentiation. Then we constructed an adenovirus Ad-BMP9 to effectively overexpress BMP9 and showed that Ad-BMP9 is capable of transfecting hAMSCs and HUVECs with high efficiency (Figure 2A). The RT-qPCR results revealed that compared with the control group, BMP9 expression in hAMSCs and HUVECs increased more than 17-fold and 12-fold, respectively, after transfection with Ad-BMP9 for 48 h (Figure 2B).

Thereafter, ALP staining was used to investigate changes in ALP activity, which is an early osteoblastic indicator. Alizarin Red S staining was to assess the alterations in bone mineralization and matrix deposition, which are late osteogenic indicators. When hAMSCs were cocultured with HUVECs, we found that ALP activity significantly increased at 3, 5, and 7 d in Group C, when compared with Group A and B; Group B exhibited the lowest ALP activity at the same time points (P<0.05). The ALP staining demonstrated that BMP9 notably increased ALP activity in Group F compared with Group D and E at 3, 5, and 7 d (Figure 2C). Quantitatively, overexpression of BMP9 in hAMSCs cocultured with HUVECs could lead to a considerable increase in ALP activity for Group D and E at 3, 5, and 7 d (Figure 2D). Alizarin Red S staining results exhibited a significant increase in bone matrix mineralization in hAMSCs cocultured with HUVECs (Group C) than Group A and B at 14 and 21 d, respectively (Figure 2E). Moreover, we found that BMP9 enhanced bone matrix mineralization and calcium deposition in Group F at 14 and 21 d, respectively, compared to other groups (Figure 2F). Collectively, the results suggest that BMP9 could greatly enhance osteoblastic differentiation of cocultured hAMSCs and HUVECs *in vitro*, suggesting that determining the incongruous expression level of BMP9 may be crucial for osteoblastic differentiation of MSCs and endothelial cells.

### BMP9 upregulates the expression of osteogenic and angiogenic-related factors of cocultured hAMSCs and HUVECs

**Figure 3:**
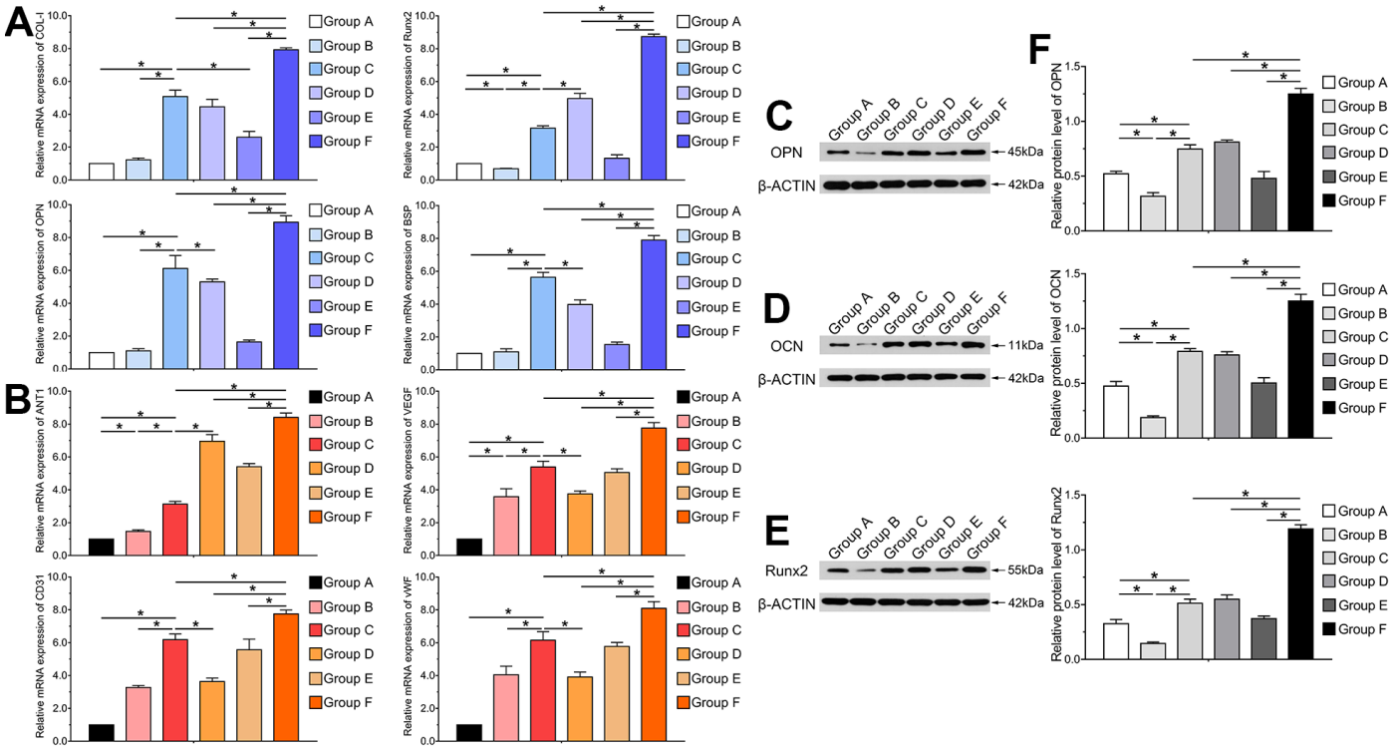
**BMP9 upregulates the expression of osteogenic and angiogenic related factors of cocultured hAMSCs and HUVECs *in vitro*.** RT-qPCR assay was performed to detect the mRNA expression level of BMP9 induced osteogenic relative factors COL-I, Runx2, OPN, and BSP in each group at 5 d (**A**); The mRNA expression level of BMP9 induced angiogenic relative factors including ANT1, VEGF, CD31, and vWF in each group at 5 d (**B**); Western blot was used to determine the protein expression of OPN (**C**), OCN (**D**), and Runx2 (**E**) of hAMSCs cocultured HUVECs at 5 d *in vitro*; Quantification analysis of Western blot showed that BMP9 significantly increased the protein expression of osteogenic relative factors OPN, OCN, and Runx2 in hAMSCs cocultured with HUVECs at days 5 (**F**) (*P < 0.05).

Furthermore, we performed RT-qPCR and western blotting analyses to investigate mRNA expression levels and protein secretion of osteogenic and angiogenic relative factors after transfection with Ad-BMP9. Runt-related transcription factor 2 (Runx2) is a primary transcription factor that acts as a critical protein regulating bone specific genes and co-regulatory proteins for osteogenesis. Runx2, OPN, OCN, collagen type I (COL-I) and binding sialoprotein (BSP) are vital indicators for osteoblastic differentiation of hAMSCs. Hence, we investigated the effects of BMP9 on gene and protein expression of Runx2, COL-I, BSP, OPN and OCN. The RT-qPCR results indicated that the mRNA expression of Runx2, OPN, COL-I and BSP was significantly up-regulated in Group F compared to other groups at 5 d (P<0.05). These factors were also significantly upregulated in Group C than Group A and B at the same time points. The mRNA expression of these factors greatly increased in Group D compared to Group A; however, BMP9 did not exert significant effects on HUVECs (P<0.05) (Figure 3A).

Our previous study suggested that BMP9 could mediate angiogenic differentiation of MSCs [29]. However, it is not clear whether BMP9 had any effects on the expression level of angiogenic factors on cocultured hAMSCs and HUVECs. Hence, we investigated the effects of BMP9 on the expression of angiogenic factors including angiopoietin 1 (ANT1), VEGF, CD31, and vWF in hAMSCs cocultured with HUVECs. Our results demonstrated that BMP9 was able to significantly upregulate the mRNA expression level of ANT1, VEGF, CD31, and vWF in Group F than in other groups at 5 d. BMP9 notably increased the gene expression level of angiogenic factors in HUVECs (Group E) than Group B. Though BMP9 upregulated the expression of these factors in Group D compared to Group A at 5 d, the upregulated level was lower than Group E and F (P<0.05) (Figure 3B).

Similarly, the western blotting and quantification results revealed that BMP9 boosted protein expression of osteoblastic relative factors OPN, OCN and Runx2 in cocultured hAMSCs and HUVECs at 5 d. The expression of OPN, OCN and Runx2 were upregulated by BMP9 significantly in Group F than with other groups (P<0.05) (Figure 3C–3F). Collectively, these results demonstrated that BMP9 exerts a positive-going regulatory effect on the expression of osteoblastic and angiogenic relative factors on cocultured hAMSCs and HUVECs.

### BMP9 reinforced the ectopic bone formation of cocultured hAMSCs and HUVECs

**Figure 4:**
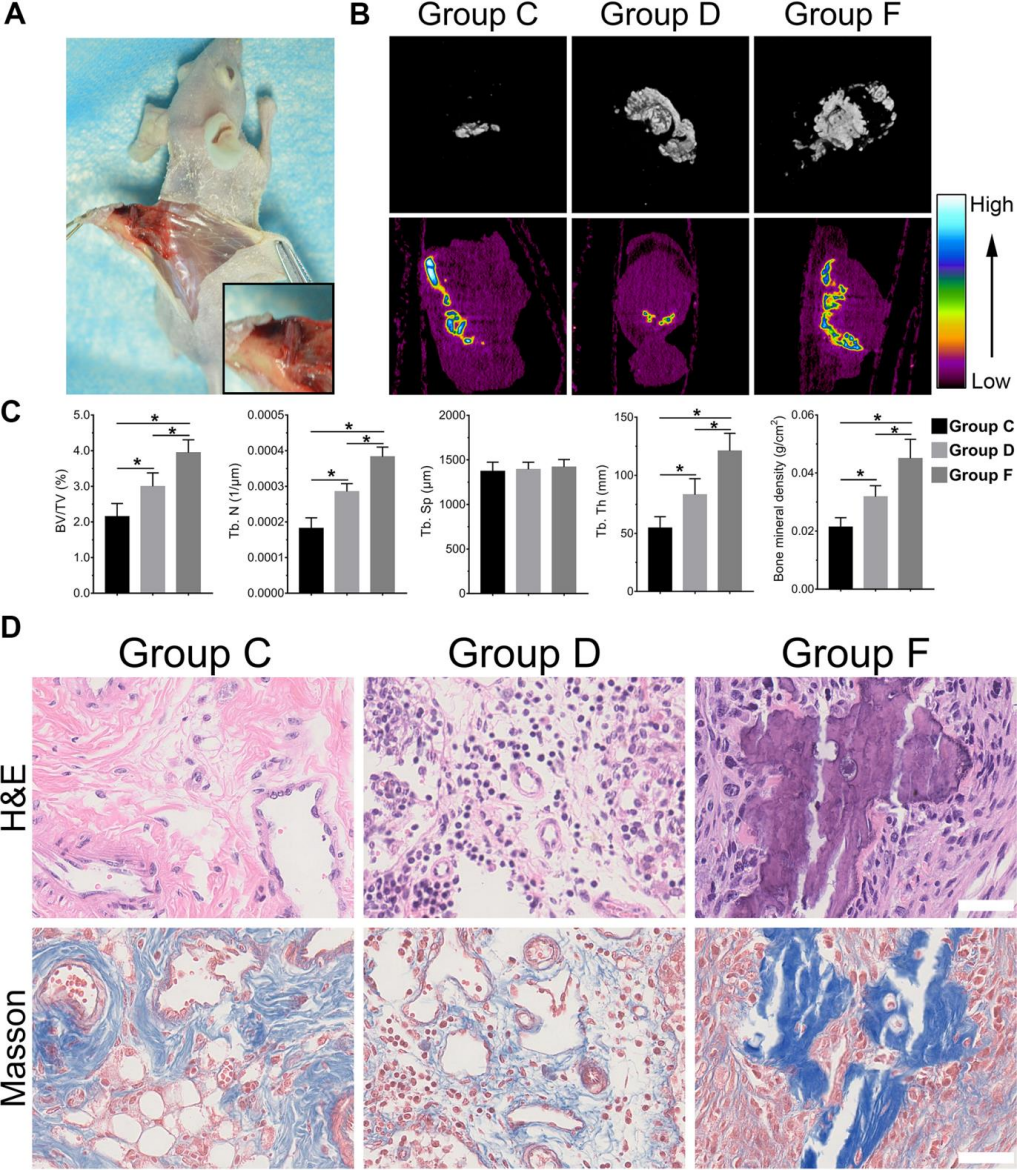
**BMP9 increases subcutaneous ectopic bone formation of hAMSCs cocultured with HUVECs.** Representative images of subcutaneous ectopic bone formation of nude mice (**A**); Subcutaneous ectopic osteogenic bone formation were detected by micro-CT scanning to investigate the 3D iso-surface and the heat map of average mineralization density at 4 weeks. In the heat map, white represents the highest average mineral density and black represents the lowest (**B**); Quantified analysis results of micro-CT scanning show the effect of BMP9 on cocultured hAMSCs and HUVECs induced ectopic bone formation, and the relative values of bone volume/total volume (BV/TV), trabecular bone number (Tb. N), trabecular bone separation (Tb. Sp), trabecular bone thickness (Tb.Th), and bone mineral density (BMD) were demonstrated (**C**); The retrieved bone masses were subjected to Trichrome-Masson staining and H&E staining to determine the formation of trabecular bone and bone matrix (×400, bar: 50μm) (**D**) (*P < 0.05).

With the above conclusion that BMP9 enhanced osteogenic differentiation of cocultured hAMSCs and HUVECs *in vitro*, we further confirmed whether BMP9 played such a role *in vivo* by ectopic bone formation in nude mice. The general observation showed ectopic osteogenesis in nude mice (Figure 4A). Size differences were detected by three-dimensional reconstruction analysis of micro-CT. The heat image analysis of mineral density revealed that BMP9 enhanced the mineral density of bone masses formed by cocultured hAMSCs and HUVECs (Group F) than other groups, yet, the average mineral density was lower in Group C than Group D (Figure 4B). The retrieved samples were further performed to histologic analysis and quantification, including values of bone volume/total volume (BV/TV%), trabecular number (Tb. N), trabecular separation (Tb. Sp), trabecular thickness (Tb. Th), and bone mineral density (BMD). These values were evidently increased in Group F as compared with Group C and D (P<0.05). There were no significant differences in trabecular Tb. Sp (Figure 4C), histologic analysis including H&E staining and Masson staining demonstrated that the number and quality of trabecular bone formation (red in H&E staining; blue in Masson Trichrome staining) in Group F increased (Figure 4D). Collectively, these results illustrated that BMP9 exerts a positive regulatory effect on osteoblastic differentiation and BMP9 augments ectopic bone formation of cocultured hAMSCs and HUVECs *in vivo*.

### BMP9 augments osteogenic and angiogenic relative factors in subcutaneous ectopic bone formation

**Figure 5:**
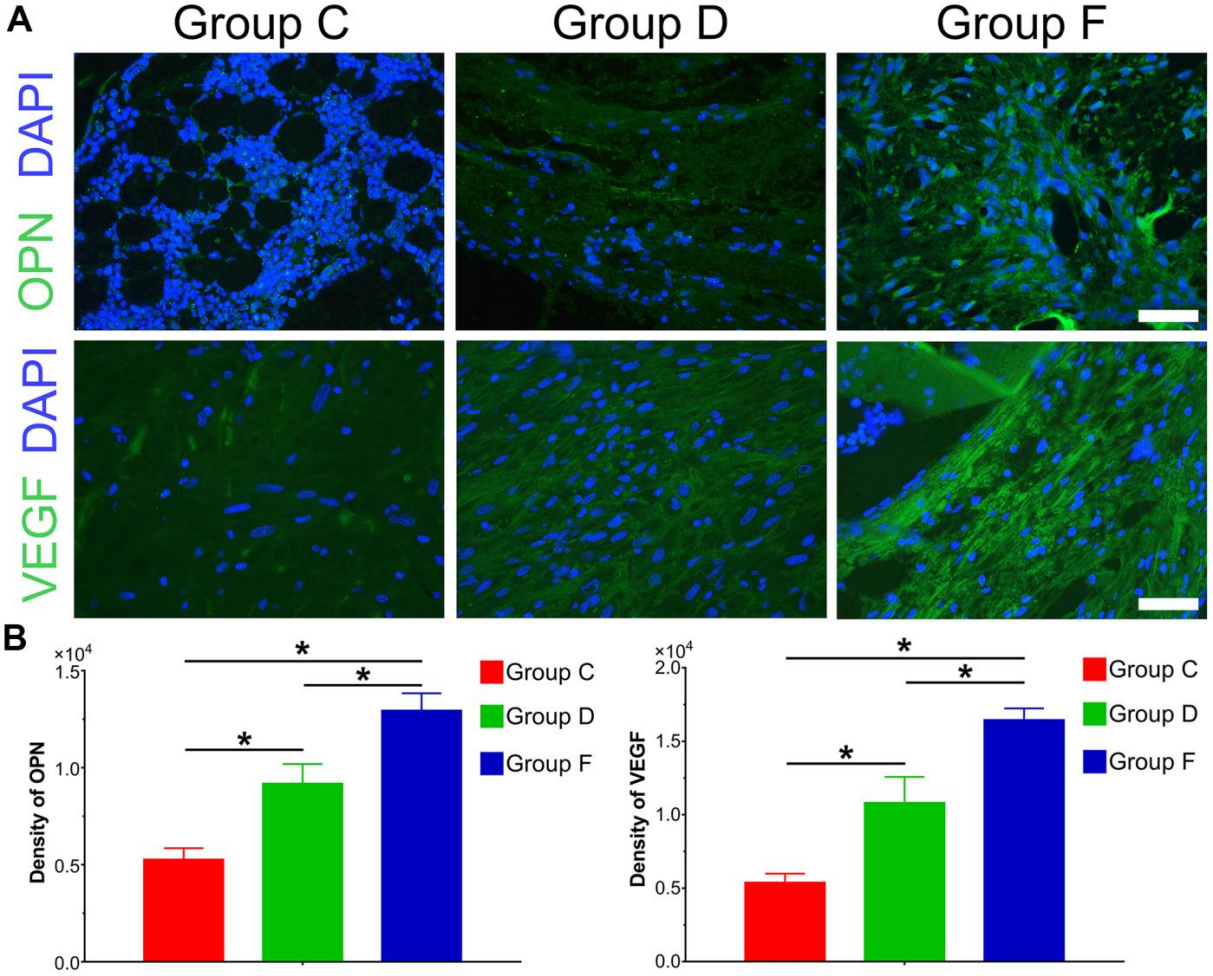
**Immunofluorescence staining and quantification analysis of osteogenic and angiogenic differentiation of cocultured hAMSCs and HUVECs *in vivo*.** Immunofluorescence and quantified analysis of ectopic bone masses to investigate the synthesis of osteogenic relative factors OPN (green) and VEGF (green) (×400, bar: 20 μm) (**A**); The protein expression of OPN and VEGF were analyzed by immunofluorescence and quantified analysis (**B**) (*P < 0.05).

The immunofluorescence results and quantification of ectopic bone formation indicated that the expression level of osteogenic relative factor OPN was significantly increased in Group F compared with control group (P<0.05). The expression level of OPN was higher in Group D than that in Group C. Furthermore, we investigated the effect of BMP9 on angiogenic relative factor VEGF. Similarly, BMP9 could potentiate the expression of VEGF in Group F as compared with Group C and D. The protein expression level of VEGF was lower in Group C than that in Group D (Figure 5A, 5B). In summary, these results suggest that BMP9 could increase protein expression of osteogenic and angiogenic relative factors in subcutaneous ectopic bone formation.

### Effects of BMP9 on vessel invasion in cocultured hAMSCs and HUVECs

**Figure 6:**
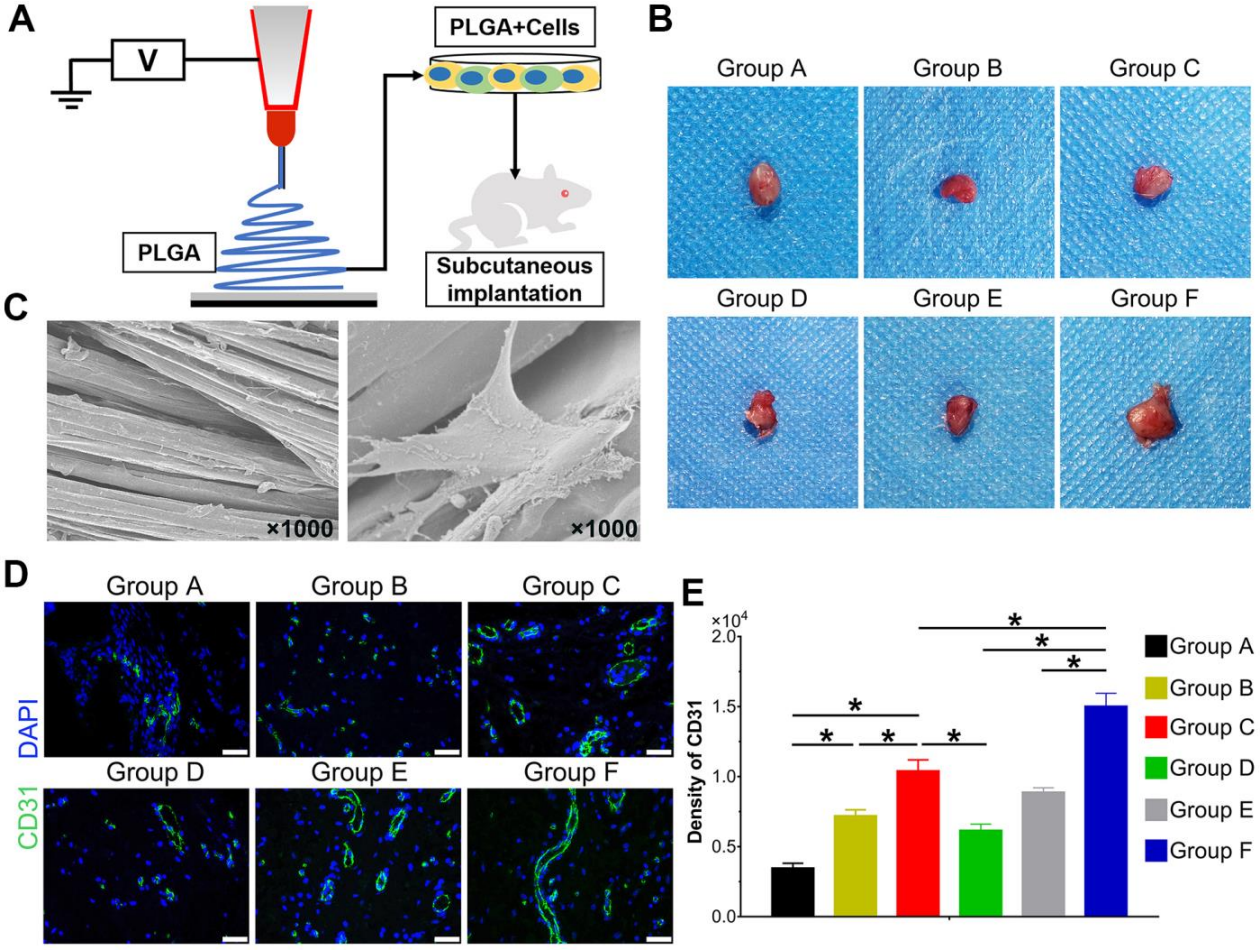
**Effect of BMP9 induced *in vitro* angiogenic differentiation and subcutaneous vascular invasion of PLGA-cells composite *in vivo*.** Illustrative diagram demonstrating cells treated according to the experimental design and seeded on electron-spun PLGA scaffold, which were implanted into the dorsal subcutaneous space of mice (**A**); Representative image of the macromorphological observation of PLGA implants located on subcutaneous tissue at 5 weeks (**B**); The topological structure of PLGA and cell-PLGA-hybrids were observed by scanning electron microscopy (SEM) (magnification ×1000) (**C**); Immunofluorescence staining and its quantification analysis were performed to detect the CD31 expression (green) in PLGA scaffold subcutaneously (×400, bar: 20 μm) (**D**, **E**) (*P < 0.05).

The hAMSCs and HUVECs were treated according to the experimental design and seeded on PLGA scaffolds for 48 h. The cells on scaffolds were implanted on the subcutaneous tissues of nude mice (Figure 6A). The topological properties of PLGA itself with cells for 24 h were detected by scanning electron microscope (SEM) (Figure 6C). After implantation for 5 weeks, mice were euthanized. The implants were retrieved and detected by immunofluorescence staining. The general graft was observed (Figure 6B) and immunofluorescence staining and its quantification data demonstrate the expression of CD31 was significantly higher in Group C than Group A and B, and Group F expressed the highest level among groups (P<0.05) (Figure 6D, E). Collectively, BMP9 not only strengthened angiogenesis of cocultured hAMSCs and HUVECs *in vitro*, it also potentiated *in vivo* subcutaneous vessel invasion ability in the cocultivation of both cells.

### The PI3K/AKT/mTOR axis mediates induced osteoblastic and angiogenic differentiation induced by BMP9 in cocultivation of hAMSCs and HUVECs

**Figure 7:**
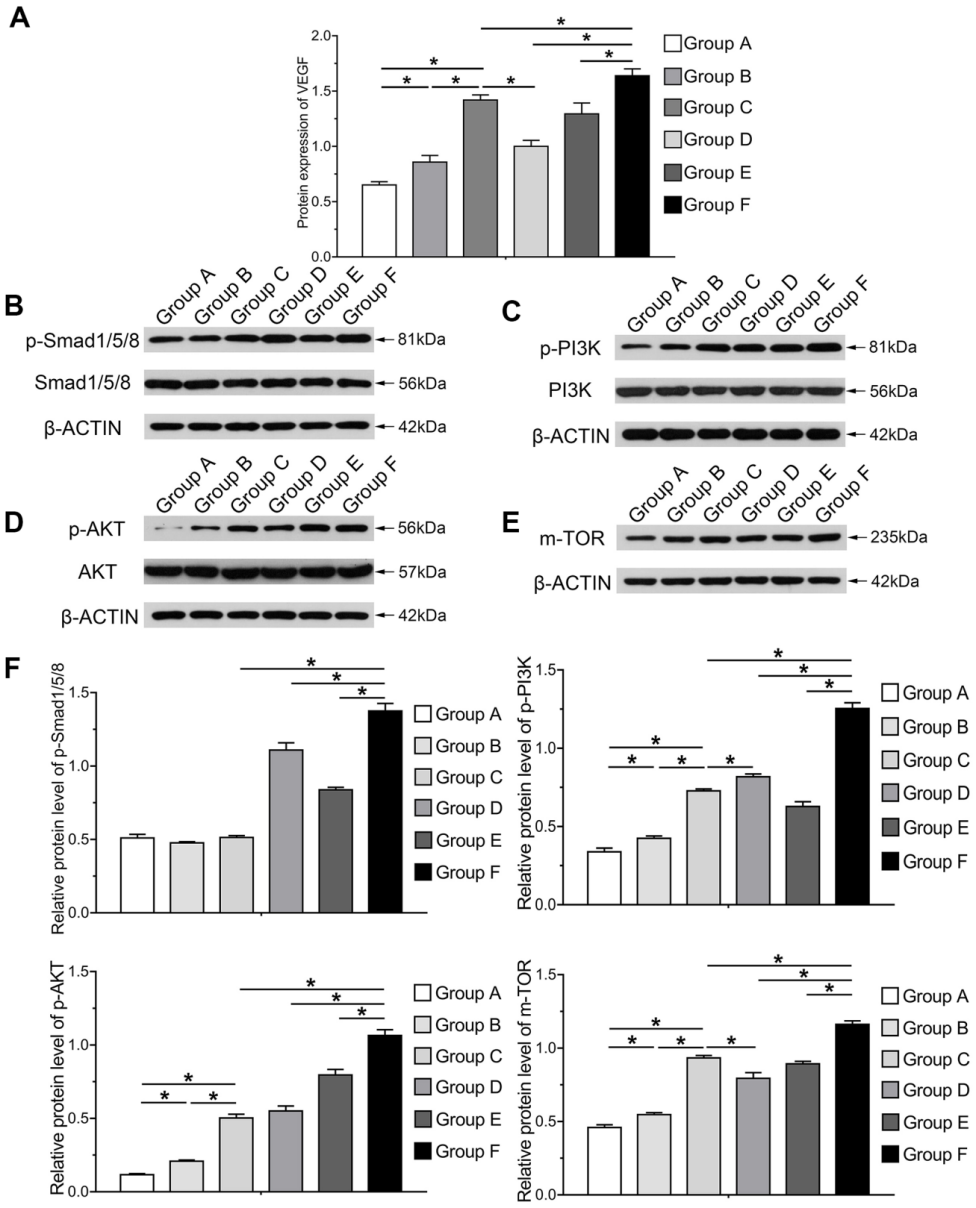
**Molecular mechanism and signaling pathways underlying BMP9 enhanced osteogenesis and angiogenesis in cocultured hAMSCs with HUVECs.** ELISA was adopted to detect the effect of BMP9 that regulates protein expression of VEGF in cocultured hAMSCs and HUVECs (**A**); Western blot and quantification analysis were used to determine the expression of p-Smad1/5/8, Smad1/5/8, p-PI3K, PI3K, p-AKT, AKT, and mTOR in each group as shown at 5 d after transfection. The results suggested that BMP9 enhanced osteogenesis and angiogenesis of cocultured hAMSCs and HUVECs by activating the BMP/Smad and PI3K/AKT/m-TOR signaling pathways through upregulating the level of phosphorylation of Smad1/5/8 and of PI3K, AKT, and protein expression of m-TOR (**B–F**) (*P < 0.05).

In order to investigate the mechanism underlying osteogenic and angiogenic differentiation induced by BMP9 in cocultured hAMSCs and HUVECs, we determined the protein expression variation of VEGF in each group induced by BMP9. Protein expression of VEGF was greatly increased in Group F than in other groups at 5 d, assessed by the ELISA assay (P<0.05). The protein level in cocultured hAMSCs and HUVECs (Group C) was higher than Group A and B (Figure 7A). The western blotting and quantification results illustrated that BMP9 notably increased the expression of Smad1/5/8 (p-Smad1/5/8) of Group F among groups (P<0.05). Compared with Group C, D and F, the p-Smad1/5/8 expression did not alert Group A, B and C without BMP9 induction. Additionally, BMP9 also increased the expression of HUVECs (Group E) than Group B. Hence, it remains clear that BMP9 on the expression of p-Smad1/5/8 in cocultured hAMSCs and HUVECs (Figure 7B, 7F).

PI3K/Akt/mTOR signaling was involved in multiple pathways and played a vital role in proliferation, migration and angiogenesis of tumor cells [30]. The mechanism by which BMP9 induces cocultured hAMSCs and HUVECs is ambiguous, so we further investigated the expression of the PI3K/Akt/mTOR.

The western blotting and quantification results demonstrated that phosphorylation of PI3K, AKT and of mTOR was increased after the induction of BMP9. The phosphorylation level of PI3K (p-PI3K), AKT (p-AKT) and mTOR in Group C was notably higher than Group A and B, which indicated that cocultured hAMSCs and HUVECs could enhance the intensity and phosphorylation level than hAMSCs and HUVECs alone. The expression level of p-PI3K, p-AKT and mTOR remarkably increased in Group F than other groups, which suggested that BMP9 could drastically up-regulate the intensity and phosphorylation level of cocultured hAMSCs and HUVECs (P<0.05). Moreover, BMP9 increased the phosphorylation of Smad1/5/8 and either p-PI3K, p-AKT and mTOR in HUVECs (Figure 7C–7F) (Supplementary Figure 1). In summary, these results may point to the effects of osteogenic and angiogenic differentiation induced by BMP9 on cocultivation with hAMSCs and HUVECs, and the mechanism may be transduced by strengthening BMP/Smad1/5/8 and BMP/PI3K/Akt/mTOR signaling pathways.

## DISCUSSION

In this study, we discussed the regulatory effect of BMP9 on osteoblastic and angiogenic differentiation in cocultured hAMSCs and HUVECs, and the possible signaling pathway underlying this process. Also we analyzed the effect of osteoblastic and angiogenic differentiation in cocultivation of hAMSCs and HUVECs. In this study, we found that cocultured hAMSCs and HUVECs could upregulate osteogenic and angiogenic markers. Additionally, we found that BMP9 can enhance osteoblastic markers, mRNAs and protein levels, while hAMSCs and HUVECs alone did not strengthen ALP activity like cells transfected with BMP9, as well as the subcutaneous ectopic bone formation and vessel invasion. We found that, mechanistically, BMP9 may exert the functions of osteogenesis and angiogenesis through intensifying BMP/Smad signaling transduction, and so does the activation of PI3K/AKT/mTOR signaling. These results strongly indicated that BMP9 exerts a crucial role in regulating osteogenic and angiogenic differentiation in cocultured hAMSCs and HUVECs, which may be initialized by regulating BMP/Smad signaling and PI3K/AKT/mTOR signaling.

hAMSCs is a group of non-hematopoietic stem cells derived from amnion tissue. These are pluripotent stem cells and can differentiate into osteoblasts, chondrocytes, and myoblasts under specific physicochemical conditions and by cytokine action [31–33]. Multi-lineages differentiation potential is one of the characteristics of hAMSCs, which is also an important reason for its stable source of seeding cells in tissue engineered bone *in vitro.* In this experiment, by using enzymatic digestion to isolate quantities of hAMSCs and HUVCEs with high purity. The cell proliferation results demonstrated that the proliferation of hAMSCs and HUVECs reached the peak at 2-3 d. Flow cytometry analysis illustrated that hAMSCs expressed the positive surface markers CD73, CD105, CD44, and CD90 and negatively expressed CD19, CD45, CD34, and HLA-DR; this is in accordance with the minimal criteria for defining multipotent mesenchymal stromal cells. It has been reported that hAMSCs expressed low or no expression of hematopoietic surface markers, immunological factor CD14, human leukocyte antigen-antigen D related (HLA-DR), which represents the main histocompatibility antigens type I including HLA-A, B, and C. Our results showed that hAMSCs have a low expression level of CD45 and HLA-DR, suggesting that hAMSCs possess low immunogenicity and immune-modulatory function [34–37]. Hence, hAMSCs can be a potential seed cells for tissue regeneration leave out immune rejection.

Tissue engineered bone is based on loading of cells with osteogenic potential, osteo-conductive scaffolds, release of osteogenic growth factors and bone tissue engineering vascularization or adequate blood supply [38, 39]. Hence, research on tissue engineered bone is currently focused on selection of excellent seeding cells and reconstruction of blood supply after implanted bone tissue engineering scaffolds. Angiogenesis has a close temporal and spatial connection with bone formation during embryonic development, bone growth and fracture healing [40]. Angiogenesis not only precedes bone formation at the temporal level, but also at the spatial level, where 80% of osteoblasts are distributed around blood vessels during bone healing [41]. Vascular endothelial cells continuously cover the intima of blood vessels, having vital physiological functions and are widely involved in physiological and pathological processes such as inflammatory response, angiogenesis, immune response, atherosclerosis, and vascular tone regulation [42, 43]. In our study, we isolated HUVECs and the immunofluorescence staining showed the HUVECs expressed the basic markers of endothelial cells. We found that hAMSCs cocultured with HUVECs can increase proliferation ability, osteogenic differentiation and bone mineralization, as well as angiogenic function including subcutaneous vessel invasion. Collectively, our results verified the stimulative effect of cocultured hAMSCs and HUVECs on osteogenic and angiogenic differentiation, and this cell-to-cell connection of cocultivation may attach great importance for regulating cell behavior and bone growth and remodeling.

BMPs are part of TGF-β super family and played an indispensable role in regulating the vital physiological destiny for cells, including proliferation, apoptosis and differentiation [44]. BMP9, also passed as growth differentiation factor 2 (GDF2), was initially discovered from the cDNA library of mouse liver [45, 46]. BMP9 has been shown to exert some effects in preserving the cholinergic phenotype of the neurons, regulation of angiogenesis, iron, stimulating hepcidin 1 expression and commitment of MSCs to osteoblastic lineage etc. [17, 47, 48]. Study have shown that BMP9 is one of the most potent regulators to induce osteoblastic differentiation among the 14 types of BMPs [19, 49]. In our study, we demonstrated that BMP9 not only upregulates the expression of osteoblastic differentiation relative factors Runx2, COL-I, BSP and OCN, it also increases the level of angiogenesis relative factors such as ANT-1, VEGF, vWF, and CD31 in mRNA as well as protein levels of cocultured hAMSCs and HUVECs. Moreover, BMP9 augments the expression of osteogenic and angiogenic factors in hAMSCs and HUVECs alone. This may suggest that BMP9 is in possession of the regulatory function of vascularization in MSCs and endothelial cells. BMP9 works its function through BMP/Smad signaling, including p38-MAPK and ERK1/2-MAPK pathway [50–52]. For the canonical BMP signaling pathway, which activates the R-Smad named phosphorylated Smad1/5/8 (p-Smad1/5/8) through binding with its membrane receptor, and then forming a complex with the phosphorylated Smad 4. The complex translocates into the nucleus and exerts its function to regulate downstream target genes [53, 54]. The results in our study shows that BMP9 increases p-Smad1/5/8 expression level of coculturing hAMSCs and HUVECs. Hence, we conclude that BMP9 enhance the osteogenic and angiogenic differentiation of cocultured hAMSCs and HUVECs by increasing the level of osteogenic and angiogenic relative factors and upregulating the BMP/Smad signaling pathway.

The osteogenic and angiogenic differentiation process is an elaborately planned progression and a number of factors take part in it, such as bFGF, BMPs, and PI3K, Akt [55, 56]. The PI3K/AKT/mTOR pathway has been shown in many types of cells including tumor cells, endothelial cells and MSCs to regulate cell functions like proliferation, angiogenesis, viability and cell migration [57, 58]. It has been reported that the PI3K/AKT/mTOR axis exerts a protective effect in tissue injury and bone development both in experimental and clinical interventions [59–61].

**Figure 8:**
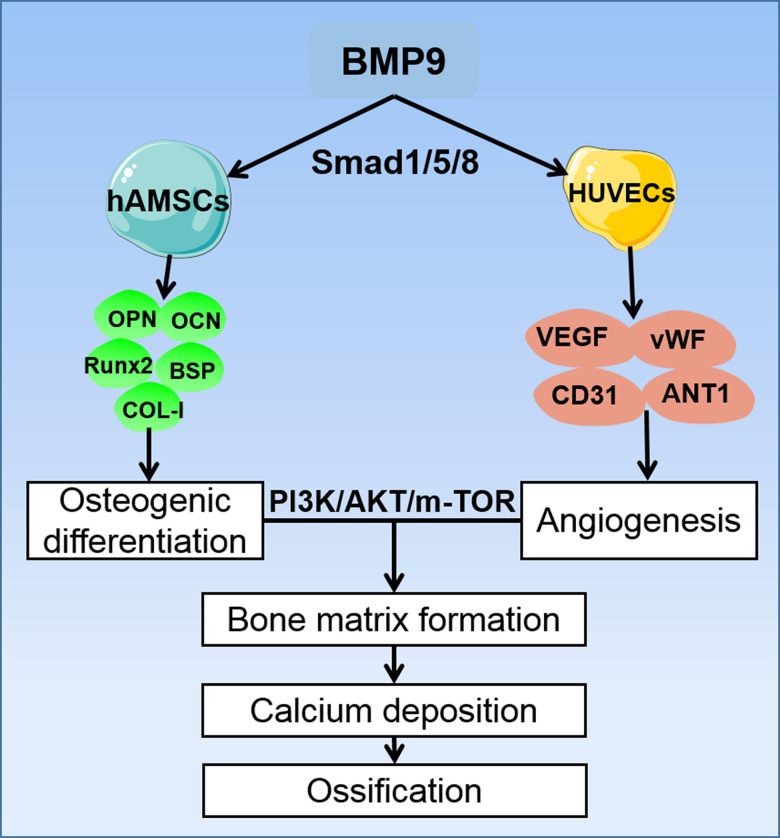
**A proposed regulatory loop of BMP9 enhanced osteogenesis and angiogenesis of hAMSCs cocultured with HUVECs.** BMP9 upregulates the osteogenic relative factors such as OPN, OCN, Runx2, BSP, and COL-I in hAMSCs, and increases the expression of angiogenic factors VEGF, vWF, CD31, and ANT-1 in HUVECs. Hence, BMP9 potentiates only osteogenic also angiogenic differentiation by coculturing hAMSCs with HUVECs. This coupling effect of osteogenesis and angiogenesis may lead to efficient bone matrix formation and ossification, and BMP9 exerts its osteogenic and angiogenic functions via BMP/Smad1/5/8 and PI3K/AKT/m-TOR signaling pathways.

Angiogenesis is a necessary condition for bone development. It involves a number of cytokines and signaling pathways, in which VEGF and its receptors bind to important signaling molecules that promote angiogenesis [62–64]. Our previous study reported that VEGF synergistically promote angiogenesis of MSCs, and current studies have confirmed that the PI3K/AKT/mTOR signaling pathway can also cause angiogenic differentiation by affecting multiple pro-angiogenic factors. In our study, the ELISA assay demonstrated that BMP9 could enhance protein expression of VEGF in cocultured hAMSCs and HUVECs and upregulated the expression level of corresponding angiogenic relative factors including CD31, vWF and ANT-1. Mechanically, by induction with BMP9, the phosphorylation of PI3K and AKT were significantly increased in cocultured hAMSCs and HUVECs. Similarly, the expression of mTOR was also upregulated by BMP9 of cocultured cells (Figure 8). Hence, the osteogenic and angiogenic differentiation of cocultured hAMSCs and HUVECs was intensified by BMP9 through BMP/Smad and the PI3K/AKT/mTOR signaling pathway.

However, there are some limitations exist in our study. Firstly, we only used the adenovirus to upregulate BMP9 expression, but no dose-effect studies were investigated. Secondly, We did not detected the osteogenic and angiogenic potential by using different cell ratios or different MOIs in this experiment, though the results do not affect the conclusion, it is still necessary to be determined in further experiment. Additionally, the role of BMP9 in the coupling of osteoblastic differentiation and angiogenesis in bone injury and regeneration remains to be further investigated.

## CONCLUSIONS

In conclusion, osteogenesis and angiogenesis are well coordinated in bone formation and rehabilitation. In this study, we demonstrated BMP9 directly enhances osteoblastic differentiation and angiogenesis in cocultivation of hAMSCs and HUVECs, which in turn, upregulate osteogenic and angiogenic factors. The coupling effect between osteoblastic differentiation and angiogenesis induced by BMP9 of cocultured cells was mainly through enhancement of BMP/Smad and PI3K/AKT/mTOR signaling pathways. Thus, favorable osteoblastic factors, including BMP9, may induce an efficaciously regulated convergence of osteogenesis and angiogenesis in cocultured hAMSCs and HUVECs.

## MATERIALS AND METHODS

### Study groups

All experiments were performed in triplicate. The hAMSCs were used as Group A and HUVECs as Group B; Group C was composed of cocultured hAMSCs and HUVECs, and Group D, hAMSCs infected with Ad-BMP9. Group E was composed of HUVECs infected with Ad-BMP9. Group F was composed of cocultures of hAMSCs and HUVECs, in which, both were infected with Ad-BMP9.

### Animals and ethics statements

This experiment was approved by the Research Ethics Committee of the First Affiliated Hospital of Zunyi Medical University. Human placentas were obtained from the Obstetrics Department of the First Affiliated Hospital of Zunyi Medical University and informed consent was provided by all patients before surgical retrieval and application. hAMSCs were isolated from five full-term puerperants, and their basic information is demonstrated in Table 1. Informed consent was provided by patients before delivery. Surgical procedures were performed on thirty-three adult male athymic nude mice (four to six weeks old, weighing 15-20 g).

**Table 1:** Characteristics of puerperants and babies providing research materials.

**Parameters**	**Mean±Std Dev**
Mother’s age (years)	25.2±1.924
Gestational age (weeks)	39±1.581
Baby sex	2F,3M
Child weight (gr)	3713±355.3

### Isolation and culture of hAMSCs

The amniotic membrane was bluntly peeled from the placenta and washed three times with phosphate-buffered saline (PBS) with 1% penicillin and streptomycin. The amniotic membrane was minced with sterile scissors into 1-2 mm^2^ sized pieces, and digested twice (30 min each) with 0.05% trypsin/0.02%. Then, the amniotic pieces were incubated with 0.75 g/L collagenase, digested at 37° C in a water bath for 60 min. hAMSCs were collected and passed through a 300-mesh filter, suspended in culture dishes containing Dulbecco’s modified Eagle’s medium (DMEM) with 10% fetal bovine serum (FBS), 1%penicillin and streptomycin, 1%L-glutamine, and nonessential amino acids. The medium was replaced every 48h, and morphology were recorded. Cells were passaged upon attaining 80% confluence, and seeded at a 1:2 ratio for subculture. hAMSCs at P3 were used for experiments.

### Phenotypic identification of hAMSCs

### Specific surface markers

In accordance with the minimal criteria for defining MSCs proposed by the International Society for Cellular Therapy (ISCT) [65], the phenotypic markers were evaluated using flow cytometry. Cells were adjusted to 2 × 10^6^ cells/mL were incubated with FITC-conjugated anti-CD90,-CD44, anti-CD105, and -CD73. Cells were combined with negative control antibodies, including PE-conjugated anti-CD34, anti-CD19, anti-CD45, anti-CD11b, and anti-HLA-DR in the dark for 30 min, washed by the addition of 2mL. The labeled cells were washed with PBS three times and resuspended in 250μL of flow buffer, and expression of surface markers was analyzed by BD Software (BD Accuri^TM^ C6 Plus Corporation, USA).

### Multilineage differentiation of hAMSCs

HAMSCs at P3 were adjusted to 10^5^ cells/mL. When cells reached 50% confluence then were cultivated in osteogenic, chondrogenic and adipogenic differentiation medium (Gibico™, USA). The osteogenic differentiation was detected by Alzarin Red S staining (Solarbio, Beijing, China). The chondrogenic differentiation was assessed by Alcian Blue staining (Solarbio, Beijing, China). Oil Red O (Solarbio, Beijing, China) staining was performed to determine the adipogenic differentiation.

### Isolation and identification of HUVECs

The umbilical cord was removed and rinsed three times in 4° C PBS with 1% penicillin and streptomycin. The hematoma was removed from the umbilical cord with PBS, then pre-heated PBS was injected to flush the blood in the umbilical vein. A hemostat was used to clamp the end of the umbilical cord and 1 g/L pre-heated type 2 collagenase was injected using a syringe into the other end of the umbilical vein to fill the lumen. The umbilical cord was placed into a petri dish containing the pre-warmed PBS, incubated at 37° C for 30 min. The digestive mixture was collected in a centrifuge tube containing 200 mL/L FBS complete medium. The cells were replaced with endothelial conditioned medium containing 10% FBS, 2 mM/L-glutamine, 1 mM sodium pyruvate, 100 U/mL penicillin, 100 μg/mL streptomycin and 1% endothelial cell growth supplement (ECGS) (ScienCell, CA, USA) after 24 h adherence, and cultured every 2 d. Immunofluorescence staining was used to identify specific markers of HUVECs, including CD31, VEGF, EMCN, and vWF.

### Cell counting kit- (CCK-8) assays

Cells were trypsinized and prepared into a cell suspension at 105 cells/mL. One hundred microliters of cell suspension was added to each well. Marginal wells were filled with sterile PBS and incubated at 37° C with 5% CO_2_. Thereafter, cells were cultured for 7 days. Ten microliters of CCK-8 solution was added to each well, the plate mixed by gentle tapping for 2 h. The absorbance was detected at 450 nm using a microplate reader, and the cell proliferation activity was calculated.

### Immunofluorescence staining

Cells were seeded on cover slips at 10^4^ cells/mL, and treated as the experimental design. Cells were washed with PBS for 10 min each, then fixed with 4% paraformaldehyde at 37° C for 15 min at the indicated time point. The cover slips were gently washed with PBS then washed with 0.4% Triton X-100 for 30 min. Thereafter cells were blocked by using goat serum for 30 min, then incubated with the first antibodies overnight. Incubation with the corresponding FITC antibodies for 60 min. Slips were washed with PBS for 10 min and stained with DAPI for 5-10 min. Results were observed by fluorescence microscope and images were assessed by Image Pro Plus software.

### Construction and infection of recombinant adenovirus

By using Ad-Easy technology as described previously, the recombinant adenoviruses were constructed [66, 67]. Briefly, the coding areas were amplified with the real time polymerase chain reaction and cloned into adenoviral vectors. The shuttle vectors were recombined with BJ5183/AdEasy-1 cells. Then, the correct recombinant vectors were linearized and transduced into HEK293 cells to package recombinant adenoviruses. The resulting adenoviruses were designated as Ad-BMP9 [68] and the Ad-BMP9 expresses GFP (green fluorescent protein). The multiplicity of infection (MOI) of transduction of hAMSCs and HUVECs was adjusted to 40% and the cell ratios of two type of cells in co-culture system was 1:1.

### ALP activity assays

Cells were adjusted into 30-40% confluence and treated as the experimental design. ALP activities were detected quantitatively by a modified Great Escape SEAP Chemiluminescence assay (Beyotime, Beijing, China) as described according to the manufacturer’s protocol [69, 70]. For the chemiluminescence assay, the results were repeated in three independent experiments.

### Alizarin S staining and mineralization assay

Cells were treated with recombinant adenovirus as the experimental design. Cells were cultivated with osteogenesis conditioned medium containing 50 mg/L vitamin C, 0.1 μmol/L dexamethasone, and 10 mmol/L β-glycerol phosphate disodium for 14 and 21 d. Mineralization was determined by Alizarin Red S staining as described previously [71, 72].

### RNA isolation and RT-qPCR reaction

Total RNA was collected to synthesize cDNA by using reverse transcription reaction kit (RR047a, Takara, Japan) following the manufacturer’s instructions. Thereafter, the cDNA were diluted 5-fold and as templates for detection by PCR. The reactions were run on a Bio-Rad CFX-96 system. All samples were normalized with the level of glyceraldehyde phosphate dehydrogenase (GAPDH). Custom-specific primers (Sangon Biotech, Shanghai, China) are as shown in Table 2. The relative expression level of mRNAs in groups was calculated using the 2^ΔΔCT^ method and was normalized to the gene expression of the control.

**Table 2:** Primer sequence of the target genes.

**Gene**	**Accession no.**	**Forward primer (5'-3')**	**Reverse primer (5'-3')**	**Length**
BMP9	NM_019506.4	GATGTTTCTGGAGAACGTGAAG	TACAGGTCAATCATGTACTGCG	111
Runx2	NM_001015051.3	AACAGCAGCAGCAGCAGCAG	GCACCGAGCACAGGAAGTTGG	183
BSP	NM_004967.4	GTCTATAGAACCACTTCCCCAC	GCTGTACTCATCTTCATAGGCT	168
COL-I	NM_000088.3	TCCGACCTCTCTCCTCTGAA	TGCTTTGTGCTTTGGGAAGT	121
OPN	NM_000582.2	AGCGAGGAGTTGAATGGTGCATAC	AATCTGGACTGCTTGTGGCTGTG	152
ANT1	NM_001146.5	GGGAGGTTGGACTGTAATACAA	TGTCATACTGTGAATAGGCTCG	209
CD31	NM_000442.5	TCGTGGTCAACATAACAGAACT	TTGAGTCTGTGACACAATCGTA	161
vWF	NM_000552.4	CCTGTTACTATGACGGTGAGAT	CATGAAGCCATCCTCACAGTAG	86
VEGF	NM_001025366.3	ATCGAGTACATCTTCAAGCCAT	GTGAGGTTTGATCCGCATAATC	132

### Western blotting analysis

Total protein was collected after cleared lysates were denatured by boiling for 10 min. Protein were separated by 8% or 10% (wt/vol) SDS-PAGE as manufacturers’ described [73]. Proteins were separated by electrophoresis and transferred onto polyvinylidene difluoride (PVDF) membranes. The PVDF membrane was blocked with 5% milk for 2 h and incubated overnight with first antibodies. Then the membranes were probed with secondary antibody. Immune-reactive signals were detected and captured using Bio-Rad. The β-ACTIN (60004-1; Proteintech) antibody was used as the internal control. Concentrations of proteins were quantified by using Image-Pro Plus software.

### ELISA

Cells were treated according to the experimental design. The cell culture medium were centrifuged to remove debris. Supernatants were diluted 5-fold. Protein level of VEGF (DVE00, R&D, MN, USA) were detected by ELISA. Standard and sample were added per well and covered with the adhesive strip and the plate was incubated for 30 min. Standard concentration holes were added as follows: 0, 1.25, 2.5, 5.0, 10.0, 20.0, 40.0, and 80.0 ng/mL, in accordance with the manufacturer’s instructions.

### Cell implantation, ectopic ossification and micro computed tomographic (Micro-CT) assay

Cells were treated with specific adenoviruses and cocultured according to the experimental design. Then the ectopic bone masses were collected for injection (5 × 10^6^ cells) from the flanks of nude mice (4-6-week old males, adult male athymic nude mice). After injection for 4 weeks, the nude mice were euthanized the samples were collected for micro-CT (Bruker Company, Belgian) and histologic staining.

Retrieved samples were decalcified for 14 days and then processed for paraffin embedding. The parameters including trabecular bone volume (BV/TV, %), trabecular number (Tb. N), trabecular separation (Tb. Sp), trabecular thickness (Tb. Th) and bone mineral density (BMD) were detected.

### Histochemical staining

The tissues were decalcified with EDTA, washed with PBS and fixed in 4% paraformaldehyde overnight at 37° C, and embedded in paraffin. Serial sections of embedded bone masses were stained with (H&E), and Masson Trichrome staining Solarbio Company, Beijing, China carried out as previously described [69, 74].

### Implantation to evaluate angiogenesis *in vivo*

Cells were seeded at 5 × 10^5^ cells/mL on PLGA scaffolds (diameter 3.5 mm, thickness 200 μm) (Lepton Company, China) for 24 h. The morphology of PLGA scaffold seeded with cells was detected by scanning electron microscopy. Eighteen mice (6 week old males; BALB/cAnN, Beijing, China), weighing 20 g, were anesthetized with 1% Pentobarbital Sodium, and the cells-PLGA compounds were implanted into the subcutaneous skin. The composite were analyzed after 5 weeks. The composite was retrieved and fixed in 4% paraformaldehyde, immunohistochemical staining was performed for CD31 (ab28364, Abcam, Cambridge, MA, USA), then by incubation with secondary FITC antibodies. The results were analyzed using an Olympus auxiliary system.

### Statistical analysis

All experiments were performed in three times. The data are reported as the mean ± SD and statistical analyses using the software package SPSS 14.0. Student q tests were used to commit significant differences. Statistical significance was set *P* < 0.05.

## Supplementary Material

Supplementary Figure 1
