# Supplementary figures and images for: Bone morphogenetic protein 9 enhances osteogenic and angiogenic responses of human amniotic mesenchymal stem cells cocultured with umbilical vein endothelial cells through the PI3K/AKT/m-TOR signaling pathway
Source: Aging (Albany NY). 2021 Nov 27;13(22):24829–49. doi: 10.18632/aging.203718 (PMC8660623; doi:10.18632/aging.203718)

SUPPLEMENTARY FIGURE

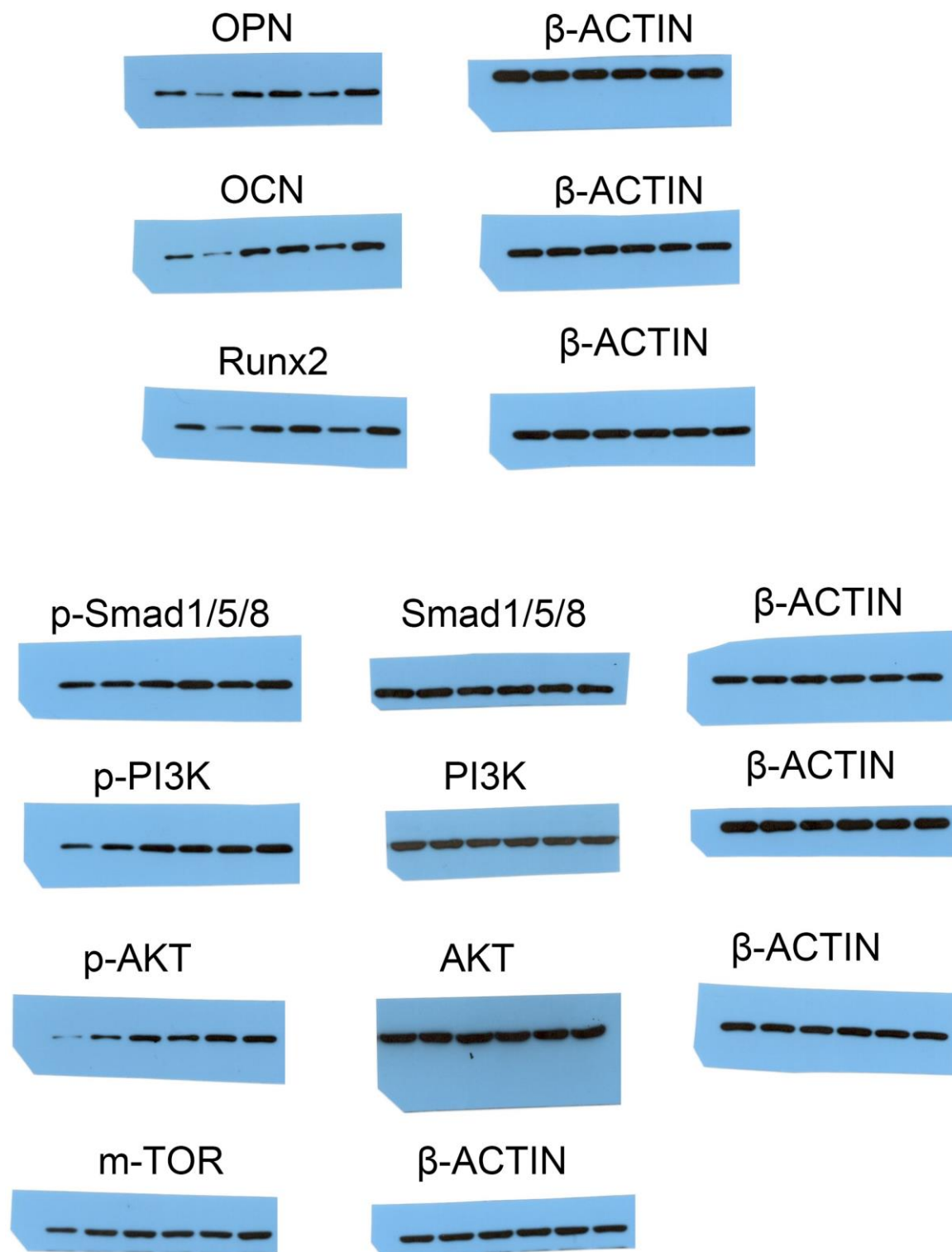

Supplementary Figure 1. The original western blots images.

Supplement: Supplementary Figure 1 [file aging-13-203718-s001.pdf]
